# Assessing genetic diversity and defining signatures of positive selection on the genome of dromedary camels from the southeast of the Arabian Peninsula

**DOI:** 10.3389/fvets.2023.1296610

**Published:** 2023-11-30

**Authors:** Mohammad Al Abri, Ahmad Alfoudari, Zainab Mohammad, Faisal Almathen, Waleed Al-Marzooqi, Salim Al-Hajri, Mahmood Al-Amri, Hussain Bahbahani

**Affiliations:** ^1^Department of Animal and Veterinary Sciences, Sultan Qaboos University, Muscat, Oman; ^2^Department of Biological Sciences, Faculty of Science, Kuwait University, Safat, Kuwait; ^3^Department of Veterinary Public Health and Animal Husbandry, College of Veterinary Medicine, King Faisal University, Al-Ahsa, Saudi Arabia; ^4^Camel Research Center, King Faisal University, Al-Ahsa, Saudi Arabia; ^5^Laboratories and Research Administration, Directorate General of Veterinary Services, Royal Court Affairs, Muscat, Oman

**Keywords:** de-correlated composite of multiple signals, environmental adaptation, signatures of selection, genetic diversity, dromedary camels

## Abstract

Dromedary camels (*Camelus dromedarius*) are members of the Camelini tribe within the Camelidae family. They are distributed throughout North Africa, the Arabian Peninsula and Southeast Asia. This domestic species is characterized by its superior adaptability to the harsh desert environment. In this study, whole autosomal data of 29 dromedary samples from the Southeast Arabian Peninsula in Oman; 10 from Muscat, 14 from Al-Batinah, and 5 from Al-Sharqiya, were investigated to assess their genetic relationship and to define candidate signatures of positive selection. A minimal genetic distinction that separates Muscat dromedaries from the other two populations was observed, with a degree of genetic admixture between them. Using the de-correlated composite of multiple signals (DCMS) approach, a total of 47 candidate regions within the autosomes of these dromedary populations were defined with signatures of positive selection. These candidate regions harbor a total of 154 genes that are mainly associated with functional categories related to immune response, lipid metabolism and energy expenditure, optical and auditory functions, and long-term memory. Different functional genomic variants were called on the candidate regions and respective genes that warrant further investigation to find possible association with the different favorable phenotypes in dromedaries. The output of this study paves the way for further research efforts aimed at defining markers for use in genomic breeding programs, with the goal of conserving the genetic diversity of the species and enhancing its productivity.

## Introduction

Dromedary camels are considered an integral part of both Bedouin and non-Bedouin societies in the Arabian Peninsula. They were indispensable means of transportation, with which owners covered vast areas of the desert for commuting and trading. These animals are patient, sturdy, able to withstand harsh environmental conditions, and can navigate through treacherous sand storms. Settlers utilized them for land plowing and transporting heavy loads over long distances. In both societies, camels provided meat, milk, hide, and a living asset that can be cashed in times of need (1). Camels still provide meat and milk today due to increasing demand for both products. However, as lifestyles and priorities in the nations of the Arabian Peninsula slowly changed with modernity, other uses for camels emerged (2). Historically, camel racing competitions have been well-known in the Arabian Peninsula. However, in modern times, racing and beauty competitions have attracted significantly more participants (3). As a result, camel owners have increasingly focused on selecting animals that excel in both racing and beauty competitions over the last few decades.

Oman’s weather is characterized by warm and dry conditions with average temperatures ranging between 16°C (in winter) and 31°C (in summer) but can soar up to 50°C during the summer (4). The exception to this weather pattern is in the southern region of Dhofar, which is affected by monsoon winds during the summer, locally known as Al-Khareef. These winds give rise to dazzle fog and occasional rain, resulting in lush vegetation and pastures (5). The Omani coastline spans over 2,000 km along the Arabian Sea and the Gulf of Oman, leading to an increase in relative humidity throughout the year in areas close to coastline (4). The average annual rainfall ranges from 50 mm in plain areas to over 300 mm in the southern region, with an overall average of about 100 mm per year (6).

Oman harbors approximately 280,000 dromedary camels, mainly used for milk production, racing or beauty competitions (7). As observed by Al Askar et al. (8), Omani camels are genetically distinct and have little to no admixture with other camel populations in the region. The majority of Omani camels, about 60%, are found in the southern region of Oman in the Governorate of Dhofar (9). It is believed that they have descended from the southern part of the Arabian Peninsula (10) and are highly regarded as some of the finest camels in the region (9), often prized for their racing abilities and stamina (8).

Assessing the genetic diversity and relationship of different dromedary camel populations was confined previously to using autosomal microsatellites (8, 11) and mitochondrial DNA markers (10, 12). Recent efforts have also utilized genotyping-by-sequencing (GBS) (13) and whole genome sequence data (14). A recent study by Almathen et al. (11) differentiated dromedary camel populations from the Arabian Peninsula into three groups utilizing 17 microsatellite loci. These groups correspond to three geographical locations: (1) North, Central, and West, (2) Southwest, and (3) Southeast. Similarly, Bahbahani and Almathen (14) observed the same geographic genetic distinctions based on whole genome sequence analyses.

Signatures of selection analyses have been performed on different livestock species, such as cattle (15–17), sheep (18, 19) and goat (20, 21), to determine candidate regions and genes associated with different favorable traits. Recent releases of the dromedary draft reference genome starting with the Arabian Peninsula (22), followed by the North African dromedary genome draft (23), and recently the chromosome-level draft by Elbers et al. (24) have all encouraged scientists to investigate the genome of dromedary camels in search for natural selection footprints (13, 25, 26). Releasing the first draft of the dromedary camel genome in 2014 revealed several gene ontologies to be under adaptive evolution, such as fat and water metabolism, response to heat stress, and salt metabolism (22). Bahbahani et al. (13) investigated the genome of dromedary racing and packing camels from Sudan for signatures of selection using genotyping-by-sequencing data where they found natural selection signals on genes associated with energy homeostasis, chondrogenesis, milk content, and immune response. Recently, a study by Khalkhali-Evrigh et al. (26) on Iranian dromedary camels also defined genes related to energy metabolism, reproduction, and long-term memory to be under natural positive selection.

Previously mentioned studies on dromedary camels relied on separate single statistical tests to detect signatures of selection. However, to improve the accuracy and resolution of detecting selection signatures, several composite analyses have been proposed that combine the signals of different statistics. These include Composite of Multiple Signals (CMS) (27), Meta-analysis of Selection Signals (28), and Composite Selection Signals (CSS) (29). While these approaches were successfully used to define candidate regions under selection in humans (27) and cattle (30), they either require accurate demographic models, as in the case of CMS, which was investigated by Fitak et al. (31), or they do not account for the covariance structure of the different single statistics employed. To address these limitations, Ma et al. (32) introduced a new approach called the de-correlated composite of multiple signals (DCMS). Compared to meta-SS and CSS, DCMS has generally shown higher power in detecting selection signatures. This approach has been previously employed to look for signatures of selection in Swedish cattle breeds (33), Russian cattle breeds (34), Russian sheep breeds (35), and Welsh sheep breeds (36).

In this study, the genetic diversity and relationship of Omani dromedary camels from the southeast of the Arabian Peninsula were assessed using whole genome sequence data. Signatures of natural positive selection were also investigated in the genome of Omani dromedary camels using the DCMS approach to defined candidate regions and genes under natural selection.

## Materials and methods

### Dromedary samples whole genome sequence data

Twenty hair bulbs were collected from each of 29 dromedary camels selected from the southeast of the Arabian Peninsula in the north of Oman: 10 from Muscat, 14 from Al-Batinah and 5 from Al-Sharqiya governorates. The samples were collected from different owners to avoid a close relationship. Hair bulbs genomic DNA was extracted using Gentra DNA purification kit as in Cook et al. (37), and sequenced using 150 bp paired-end libraries on an Illumina Hiseq 2000 platform at IGA Technology Services (Udine, Italy).

### Whole genome sequence data processing and variants calling

The adaptor-free sequence reads were mapped against the Arabian dromedary camel reference genome assembly (GCF_000803125.2) using the *bwa-mem* algorithm of Burrows-Wheeler Aligner (BWA) version 0.7.17 (38). Reads were sorted by coordinates using the *SortSam* algorithm and SORT_ORDER = coordinate option, and duplicates were marked and excluded using *MarkDuplicates* algorithm and REMOVE_DUPLICATES = true option in Picard tools version 3.0.0.^1^ Summary statistics calculated for mapped reads included: the proportion of reference genome covered, mean depth of coverage, and percentage of mapped reads via the *coverage* and *flagstat* tools in SAMTools software version 1.13 (39).

Single Nucleotide Polymorphisms (SNPs) were called across all samples using the *HaplotypeCaller* tool in *GVCF* mode of GATK version 4.2.5.0 (40). The variants were subsequently combined and genotyped using the GATK *CombineGVCFs* and *GenotypeGVCFs* tools resulting in a total of 8,010,983 SNPs. After selecting autosomal SNPs, variants were hard-filtered using the Var*iantFiltration* tool of GATK to exclude: (1) variants with high probability strand bias between reference and alternate alleles (FS > 60); (2) variants with low quality by depth (QD < 2); (3) variants with a low root mean square mapping quality (MQ < 40); (4) variants with low phred-scaled variant probability (QUAL < 30); (5) variants with strand bias in mapping quality between reads supporting reference or alternate alleles (MQRankSum < −12.5); and (6) variants where the position of the alternate allele exhibits a bias toward the ends of the reads (ReadPosRankSum < −8). SNPs with a depth of coverage ranging between two reads and three standard deviations from the mean depth of coverage across samples were retained to end up with a total of 5,099,313 autosomal SNPs.

### SNPs quality control pruning

The retained autosomal SNPs underwent two separate quality control pruning criteria using PLINK v1.9 (41) for each of the genetic diversity and signature of selection analyses. For the diversity analysis, autosomal SNPs were pruned if: (1) their call rate was <100% of the genotyped samples; (2) they departed from the Hardy–Weinberg equilibrium (*p-*value <1 × 10^−6^); or (3) they had a minor allele frequency (MAF) ≤5%. Linkage disequilibrium pruning was also implemented using the PLINK option (*--indep-pairwise 50 10 0.5*), as in Ming et al. (42), to exclude SNPs with a correlation coefficient (*r*^2^) > 0.5. Average *r*^2^ was calculated between SNP pairs using the PLINK option (--r2 --ld-window 1000000 --ld-window-kb 2000 --ld-window-r2 0.09) (Supplementary Figure S1). The same quality control criteria were applied to SNPs for the signatures of selection analyses except for linkage disequilibrium, which is considered as a signal of selection. The final number of SNPs for the genetic diversity and signatures of selection analyses remaining were 208,524 and 3,138,930, respectively (Table 1). Samples were filtered out if they had a genotyping call rate <100% or a maximum pairwise identity-by-state (IBS) ≥95%, in which case the sample with the lower call rate was excluded. No samples were excluded due to these criteria.

**Table 1:** Quality control criteria and the number of excluded and remaining SNPs for the genetic diversity and signatures of selection analyses.

Dataset		Number of SNPs	
Raw autosomal SNPs		5,099,313	
Quality control criteria		Number of excluded SNPs	
Diversity analyses	Signatures of selection analyses	Diversity analyses	Signatures of selection analyses
Genotypic call rate < 100%	Genotypic call rate < 100%	851,275	851,275
MAF ≤ 5%	MAF ≤ 5%	1,039,085	1,039,085
HWE (*p*-value <1 × 10^−6^)	HWE (*p*-value <1 × 10^−6^)	70,023	70,023
Linkage disequilibrium (*R*^2^ > 0.5)		2,930,406	
Final number of SNPs		208,524	3,138,930

### Genetic diversity analyses

Observed homozygosity and inbreeding coefficient (*Fis*) were computed for the different dromedary camel populations using the *hom* function of the GenABLE package (43) in R software version 4.1.0 (44). Two-sample Mann–Whitney U test was used to test for statistically significant differences in the homozygosity and *Fis* values between different dromedary populations. One-sample Mann–Whitney U test was used to check if the *Fis* values of each of the dromedary populations were significantly different from zero. Principal Component Analysis (PCA) was conducted on the filtered SNP data to determine the genetic relationship between the dromedary populations. *Prcomp* function implemented in R software was used to define the different principal components and the amount of variation explained by each component. The first two components were plotted using the ggplot2 package (45) of R software. Local ancestry proportions of the different dromedary samples were estimated using the ADMIXTURE 1.23 software (46). Ancestral cluster (*K*) values ranging from 1 to 3 were assumed to reflect the total number of populations in the dataset. A total of 200 bootstrap iterations were performed for each *K* analysis. The *K* cluster with the lowest cross-validation error (cv) was considered as the optimal number of clusters fitting the dataset.

### Signatures of selection analysis

The de-correlated composite of multiple signals (DCMS) approach (32) was used to detect signatures of selection on the autosomes of the dromedary camels. This approach employed four statistical tests: two allele-frequency spectrum-based statistics [Tajima’s D index (47) and nucleotide diversity (pi) (48)]; and two intra-population haplotype-based statistics [integrated haplotype score (iHS)] (49) and number of segregating sites by length (nSL) (50).

Tajima’s D statistic was estimated in sliding 100 kb windows with a 25 kb step using the *Tajima* flag implemented in the VCF-kit tool version 0.2.6 (51). Nucleotide diversity (pi) was calculated for each chromosome separately on a per-site basis using the *--site-pi* function implemented in VCFtools version 0.1.16 (52). For calculating iHS and nSL scores, Beagle (53) was used in its default parameters for haplotype phasing to determine the individual haplotypes. After haplotype phasing, the iHS and nSL scores were obtained for every SNP using the selscan tool and default parameters (54). Before combining the four statistics, the nucleotide diversity, iHS, and nSL values were all standardized individually in sliding 100 kb windows with a 25 kb step and yielded mean values using an in-house R script.

In the final step, the 100 kb windows of the four statistics were all processed using the MINOTAUR package (55) in R software. After excluding windows with less than 10 SNPs, the results of the four statistics were converted to *p-*values based on fractional ranks using the *stat_to_pvalue* function. The *p-*values were then transformed to rank-based *p-*values based on either the one-tailed test (Tajima’s D and pi - left-tailed) or two-tailed tests (iHS and nSL). Then, the correlation between the four statistics was calculated by constructing a covariance matrix using the *Cov-NAMcd* function with *α = 0.75* and *nsamp = 50,000*. This matrix was then used to adjust for correlation among the statistics and obtain the DCMS values concurrently with the *DCMS* function. *p-*values for the DCMS values were calculated using the *pnorm* function in R. Windows with –log_10_ (*p-*values) ≥ 4, which is equivalent to *p-*values ≤0.0001, were defined as candidate windows with signatures of selection. Finally, overlapping windows were merged into a single candidate region under selection.

### Functional annotation of the candidate signatures of selection regions

The coordinates of the candidate regions were cross-referenced against the dromedary camel reference genome assembly (GCF_000803125.2) genes list using the *GenomicRanges* package (56) in R. Functional profiling of the overlapping genes was conducted using the (g: GOSt) function of the *gProfiler* web server (57), which determined the functionally enriched terms for the Gene Ontology (GO), biological processes and molecular functions. The gprofiler (g: SCS) algorithm was used to compute multiple testing corrections for *p*-values from GO and pathway enrichment analyses. All the identified genes were also processed using the functional annotation enrichment tool implemented in *DAVID* Bioinformatics resources 6.7 (58) to determine enriched functional terms. An enrichment score of 1.3, which is equivalent to the Fisher exact test *p-*value of 0.05, was used as a threshold to define the significantly enriched functional terms in comparison to the dromedary reference genome background. The genes were then cross-referenced with literature to evaluate their biological functions. Genomic variants in the candidate regions were annotated with SnpEff software version 4.3 (59).

## Results

### Summary statistics of mapped sequence reads

The depth of coverage of the mapped sequence reads among the Omani dromedary samples ranged from 7.93X to 22.5X with a mean of 11.4X (Supplementary Table S1). On average, 99.8% of the sequence reads were mapped to the dromedary reference genome, out of which 96.2% of them were properly paired. These mapped reads covered an average of 94.8% of the reference genome (Supplementary Table S1).

### Genetic diversity analyses

The mean observed homozygosity values were estimated as 0.576 for Muscat, 0.575 for Al-Batinah, and 0.563 for Al-Sharqiya dromedaries, which were not significantly different from each other (*p-*value > 0.05). Negative *Fis* values were calculated for the different dromedary camels: −0.062 for Muscat, −0.062 for Al-Batinah, and −0.093 for Al-Sharqiya camels. The different *Fis* values were not significantly deviated from zero and did not show significant differences among each other (*p-*value > 0.05).

**Figure 1:**
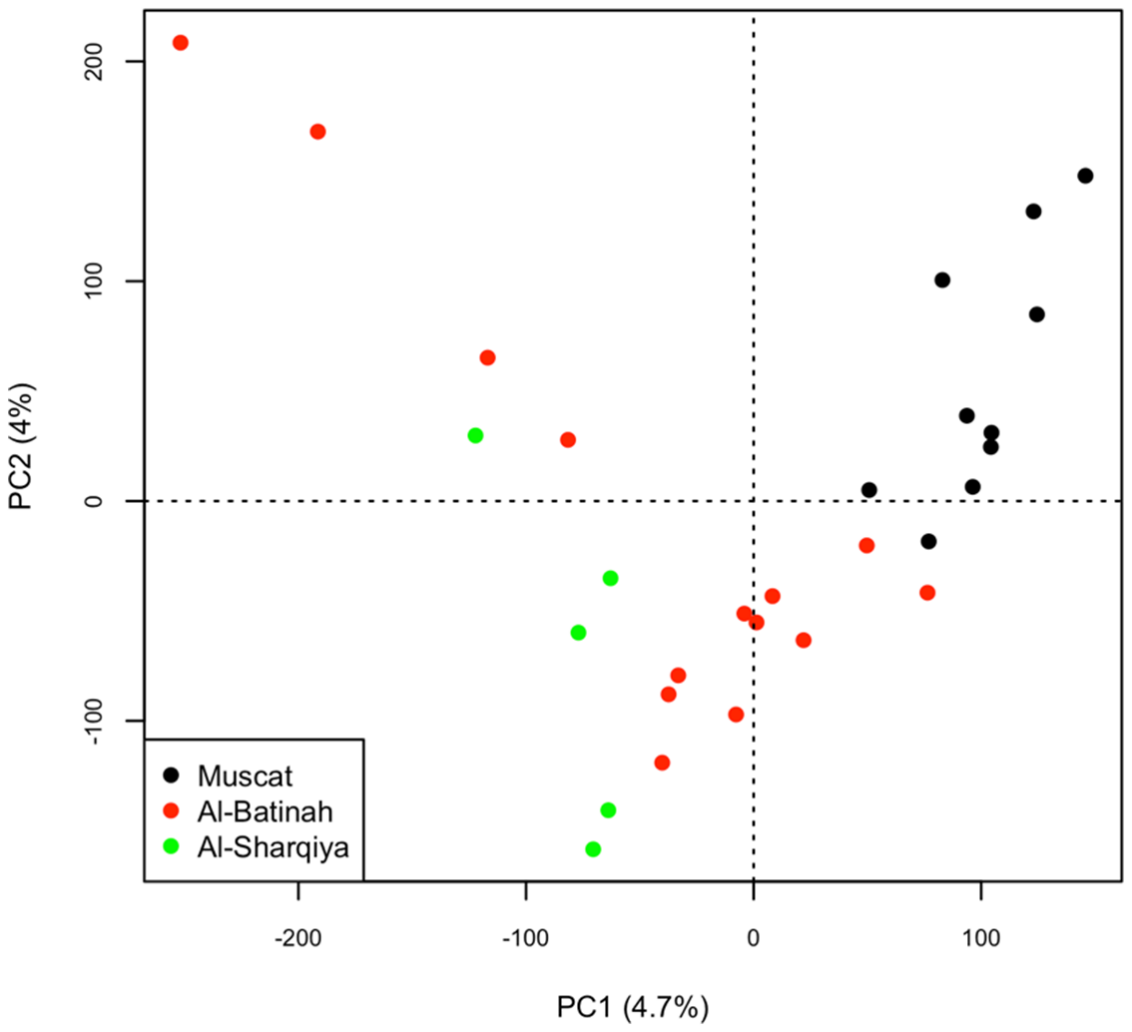
Principal Component Analysis (PCA) plot on the Muscat, Al-Batinah and Al-Sharqiya dromedaries.

The principal component analysis showed a degree of genetic separation between the Omani camels from Muscat and the other Omani camels through the first principal component, which explained 4.7% of the total variation. Along this component, the Omani camels from Al-Sharqiya were slightly separated from Al-Batinah camels (Figure 1).

**Figure 2:**
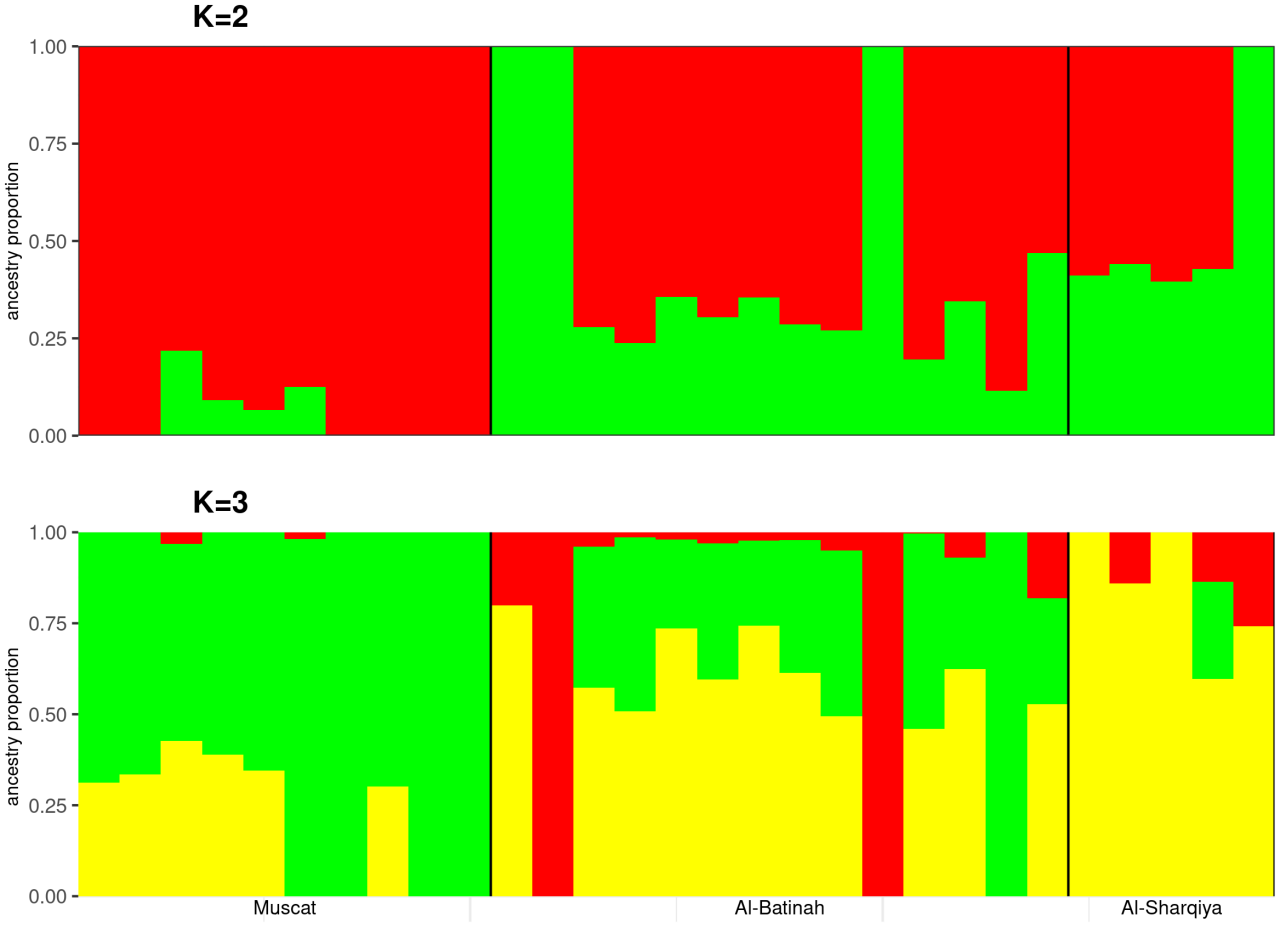
Admixture analysis plots of *K* = 2 and 3 on the Muscat, Al-Batinah, and Al-Sharqiya dromedaries.

The optimal number of clusters determined by the admixture analysis was *K* = 1 (Supplementary Table S2). At *K* = 2, a genetic ancestry background related to Omani camels from Muscat was observed. A substantial degree of genetic admixture also observed among the Omani dromedaries analyzed at all *K* values tested (Figure 2).

### Signatures of selection analysis

**Figure 3:**
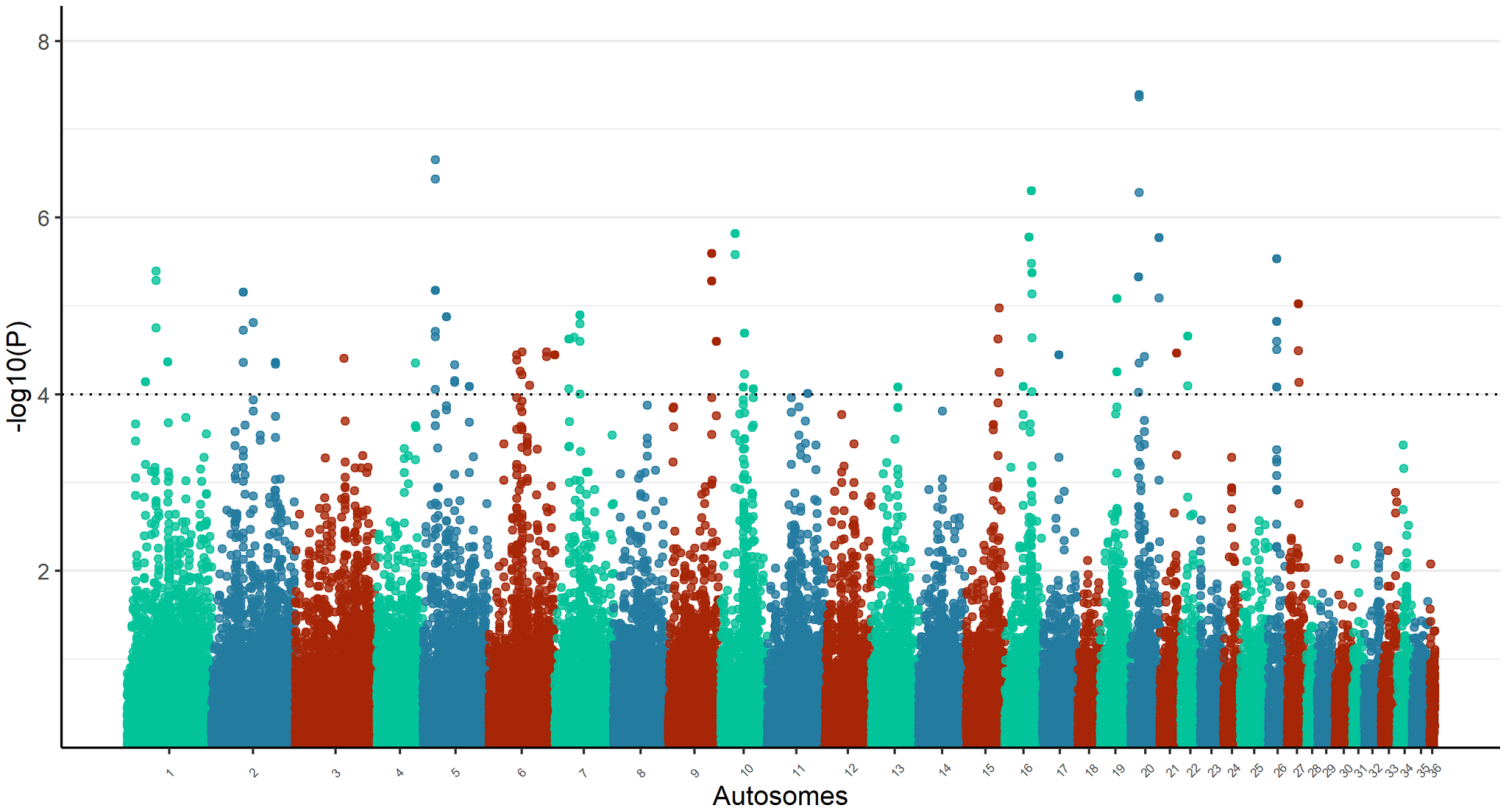
Manhattan plot of DCMS analysis on the autosomes of Omani dromedary camels. The horizontal line represents a significant *p-*value threshold of 1 × 10^−4^.

The calculated DCMS values for the total 77,133 windows on the genome of the Omani dromedary camels ranged from −14.74 (*p-*value = 1) to 14.67 (*p-*value = 4.04 × 10^−8^), in which a total of 87 candidate windows passed the threshold of *p-*value <1 × 10^−4^ (Figure 3, Supplementary Table S3). The candidate windows were merged into 47 regions ranging in size from 100 kb to 225 kb with a mean size of 123.94 kb ± 32.54 kb. These regions were distributed among 20 autosomes with chromosome 6 having the highest number of regions (*n* = 6) (Supplementary Table S4). The largest candidate regions were in chromosomes 20 (117.5 Mb to 119.8 Mb) and 7 (209.0 Mb to 211.3 Mb), while the most significant candidate window was in chromosome 20 (118.3 Mb to 119.3 Mb) (Supplementary Table S4).

### Functional profiling of the candidate regions

A total of 154 genes were found within the 47 candidate regions relating to different functional categories, such as immune response, lipid metabolism and energy expenditure, optical function, long-term memory, and fertility (Table 2, Supplementary Table S5). The functional profiling of the defined genes indicated different functionally enriched molecular and biological processes relating to molecular adaptor activity, dopamine signaling, olfactory behavior, and Wnt signaling (Supplementary Table S6). Based on the DAVID analysis, eight functional clusters were identified, showing enrichment for functions relating to transcription (enrichment score = 1.04), diseases (enrichment score = 0.8), Wnt signaling pathway (enrichment score = 0.66), beta-transducing repeats (enrichment score = 0.58), acidic and basic amino acids (enrichment score = 0.44), extracellular regions (enrichment score = 0.36), immunoglobulin domains (enrichment score = 0.35), and transmembrane helices (enrichment score = 0.26) of which none were significantly enriched (Supplementary Table S7). A total of 12,089 variants were called on the defined candidate regions, which were classified into different types and the majority of them being intronic variants (59.5%), followed by intergenic region variants (16.67%), downstream gene variants (10.08%) and upstream gene variants (7.89%) (Table 3, Supplementary Table S8).

**Table 2:** Examples of candidate genes and their number of variants in different functional categories within the candidate regions.

Functional category	Gene ID	Candidate region (Chr: start-stop)	Total number of variants
Immune response	*GMFG*	9:64.00 Mbp-64.17 Mbp	8
	*IL17RD*	17:23.72 Mbp-23.82 Mbp	133
	*SLC22A7*	20:11.75 Mbp-11.97 Mbp	40
Lipid metabolism and energy expenditure	*SELENOV*	9:64.00 Mbp-64.17 Mbp	11
	*STEAP2*	7:20.90 Mbp-21.12 Mbp	31
	*NBEAL1*	5:68.40 Mbp-68.50 Mbp	109
Optical function	*RLBP1*	27:16.52 Mbp-16.67 Mbp	47
Long-term memory	*SYT3*	9:70.90 Mbp-71.00 Mbp	64
	*TTBK1*	20:11.75 Mbp-11.97 Mbp	142
Auditory function	*CRIP3*	20:11.75 Mbp-11.97 Mbp	32
	*SLC22A7*	20:11.75 Mbp-11.97 Mbp	40
Fertility	*CFAP69*	7:20.90 Mbp-21.12 Mbp	32
	*SRF*	20:11.75 Mbp-11.97 Mbp	32

**Table 3:** Types, counts, and percentages of genomic variants called in the candidate regions.

Type of variants	Count	Percentage
Intron variant	17,514	59.50%
Intergenic region	4,908	16.67%
Downstream gene variant	2,967	10.08%
Upstream gene variant	2,322	7.89%
Missense variant	409	1.39%
3-prime UTR variant	361	1.23%
Synonymous variant	358	1.22%
Noncoding exon variant	239	0.81%
Intergenic variant	133	0.45%
5-prime UTR variant	117	0.40%
5-prime UTR premature start codon gain variant	40	0.14%
Splice region variant. Intron variant	35	0.12%
Nonsense variant	16	0.05%
Missense variant. Splice region variant	7	0.02%
Splice region variant	6	0.02%
Splice acceptor variant. Intron variant	1	0.01%
Splice region variant. Noncoding exon variant	1	0.01%
Splice region variant, synonymous variant	1	0.01%

## Discussion

The analyzed Omani dromedary camel populations showed a minimal degree of genetic distinction mainly related to their distinct geographical origins: Muscat, Al-Batinah, and Al-Sharqiya. Such genetic distinction might also be related to the different types of dromedaries analyzed, as dromedary camels from Muscat are known as production camels while camels from Al-Batinah and Al-Sharqiya are non-production type, i.e., beauty and racing types. According to camel owners, Omani production camels are rarely interbred with beauty or racing camels. Conversely, limited interbreeding occurs between beauty and racing camels. This type-wise genetic differentiation needs to be further validated by sequencing more dromedary samples from these different classifications.

A substantial level of genetic introgression was also observed among the Omani dromedary populations, which may reflect the historical use of dromedaries in transportation and trading linking the different parts of the Arabian Peninsula through the “Incense Route” (60). Similar genetic phenomena have also been observed in dromedary camels from the Arabian Peninsula based on autosomal microsatellite data (11), whole genome sequence data (14), and in African dromedary camels (13). The practice of outbreeding among camel owners, characterized by random breeding between different camel populations, could also contribute to the observed genetic admixture. This is supported by the mean negative *Fis* values calculated for the different dromedary populations analyzed.

Several candidate genome regions were defined with signatures of positive selection based on the DCMS approach on Omani dromedary camels. This approach has the advantage of combining the signals of the different signature of selection statistics and outperforms the power of any single statistic (32). The use of such composite statistics, as seen with meta-SS, CMS, and CSS, will improve the resolution of localizing the selection hotspot (27, 28, 32).

The defined candidate regions harbor genes mainly related to different signaling pathways, such as Wnt signaling and dopamine signaling, immunity, hematopoiesis, fat metabolism and energy expenditure, and thermoregulation. These biological functions might be related to the adaptations of dromedary camels within their surrounding environment and habitat. The Wnt signaling pathway, a functionally enriched pathway identified among the genes in the candidate regions, has a main role in myogenesis. The canonical and non-canonical Wnt signaling pathways are involved in regulating the differentiation of muscle stem cells and the growth of skeletal muscle fibers, respectively, which have a significant impact on the ability of dromedary camels to endure long-distance transportation along the sandy deserts (61).

Genes with biological roles associated with immunity, such as glia maturation factor gamma (*GMFG*) interleukin 17 receptor D (*IL17RD*), and solute carrier family 22 member 7 (*SLC22A7*) were found within three candidate regions on chromosomes 9, 17, and 20, respectively. These biological functions are considered natural selection hotspots in dromedaries, as they enable them to tolerate infections and pathogen loads in the desert environment. The *GMFG* gene plays an important role in maintaining effective cellular immunity. The encoded protein is a component of T-lymphocytes pseudopodia and is hence involved in regulating their migration (62). Interleukin 17 receptor D is involved in controlling inflammation upon mediating IL-17A-induced proinflammatory gene expression as observed in keratinocytes and psoriasis-like skin inflammation (63). *IL17A* has also been found to play a role in tissue repair by enhancing cellular adaptation to chronic hypoxia upon activating hypoxia-inducible factor 1a (HIF1a) pathway in epithelial cells (64). *SLC22A7* is an organic anion transporter involved in regulating blood uric acid level by the renal systema (65). Uric acid plays a role in triggering interleukin-mediated inflammation and induction to type 2 immune response (66).

Energy and fat metabolism, as well as visual system, were found to be under adaptive evolution in camelids by Wu et al. (22). Genes related to these functional categories have also been found in the defined candidate regions in this study. The Selenoprotein V (*SELENOV*) gene in the candidate region (9: 64–64.17 Mb) is an example of a gene involved in fat metabolism and energy expenditure. In a mouse knockout experiment by Chen et al. (67), high-fat mass accumulation and a decrease in energy release have been observed upon *SELENOV* depletion. *STEAP2* gene in the candidate region (7: 20.9–21.1 Mb) is another example of a gene related to fat metabolism. This gene has been found to be significantly up-regulated in myogenic precursors, which differentiates skeletal muscle fibers. The balance between myogenesis and adipogenesis during skeletal muscle development is related to intramuscular fat content (68). Neurobeachin like 1 (*NBEAL1*) gene in the candidate region (5: 68.4–68.5 Mb) is involved in cholesterol metabolism. The encoded *NBEAL1* protein regulates the expression of low-density lipoprotein (LDL) receptors, which are required to uptake extracellular cholesterol from LDL upon controlling the sterol regulatory element-binding protein 2 (*SREBP2*) processing (69). *NBEAL1* has also been involved in regulating body temperature in cattle, as highlighted in by Howard et al. (70). Interestingly, this gene has been considered as a candidate of selection pressure in locally adapted Mediterranean sheep (71), Ethiopian sheep (72), and Ugandan goats (73). Long-term ultraviolet radiation exposure is a selective force facing dromedary camels in the desert environment, which may target genes related to photoreception, such as the *RLBP1* gene found in the candidate region (27: 16.5–16.67 Mb). This gene codes for the cellular retinaldehyde-binding protein (*CRALBP*) in the retinal pigment epithelial and Muller cells of the retina. *CRALBP* is involved in the visual cycle (retinoid cycle) for continued light detection by rod and cone photoreceptor cells (74, 75).

Dromedary camels are characterized by a long-term memory that was commonly used to remember the routes of long journeys, hence genes related to neural development and differentiation may be considered as targets of selection. Synaptotagmin 3 (*SYT3*) gene was found in a candidate region on chromosome 9 (9: 70.9–71 Mb). The synaptotagmin 3 protein is an integral membrane protein localized in the postsynaptic endocytic zone of neurons. This protein is important to promote forgetting, which is crucial to maintain an acquired memory in changing environments. A study conducted by Awasthi et al. (76) demonstrated a lack of forgetting ability in *SYT3* knock-out mice. Tau-tubulin kinase 1 (*TTBK1*) found in the candidate region (20: 11.75–11.97 Mb) is another example of a gene related to neural development and memory. This gene has been found to encode neuron-specific protein that regulates the phosphorylation of the tau protein. Tau protein hyperphosphorylation and aggregate formation are correlated to various neurodegenerative disorders, such as Alzhiemer’s disease, and dementia across several species (77).

The desert environment has a significant impact on the physical properties of sound, with factors such as humidity-related attenuation and sound propagation altering the way that sound travels. Unfortunately, these changes can also make dromedary camels vulnerable to abiotic noise, especially wind noise, which can disrupt their auditory awareness and balance (78). Genes related to hearing function have also been found in this study, particularly the genes *CRIP3* and *SLC22A7* both found in chromosome 20 (20: 11.75–11.97 Mb). Variants on the genes *CRIP3*, which plays a role in T-cell proliferation and metal-ion binding, and *SLC22A7*, have been found to be associated with hearing loss in humans (79).

A major challenge facing dromedary camels is their reproductive fitness in such a harsh desert environment. Therefore, genes involved in spermatogenesis, such as the *CFAP69* gene in the candidate region (7: 20.9–21.1 Mb), are a target of natural selection to maintain optimal sperm motility. This gene, which codes for cilia and flagella-associated proteins, has been found to be associated with multiple morphological abnormalities of sperm flagella (MMAF) syndrome and consequently male infertility. Knocking out this gene in mice revealed severe disruption to the sperm flagellum structure (80). The serum response factor (*SRF*) gene in the candidate region (20: 11.75–11.97 Mb) may also be another target of selection. This gene has been found to play a role in early embryonic development and its knockout leads to embryonic lethality by mid-gestation (81).

Several uncharacterized loci were found within the defined candidate regions, more specifically in chromosome 6 (6:38.57–38.7 Mb), which is one of the most significant regions defined in this study. These loci need to be further investigated to determine their biological roles that might be related to the dromedaries’ adaptability. In addition to these loci, the most recent draft of the dromedary genome (CamDro3) contains 21,032 scaffolds unmapped to the autosomes or sex chromosomes and consequently were excluded from the analyses here. The genes on these scaffolds can be considered as potential targets of selection. Further sequencing efforts are required to map these DNA elements into their corresponding chromosome to be investigated for signatures of selection.

Different classes of genomic variants have been identified in the candidate regions, in which some of them are associated with functional impacts: such as amino acid changes, i.e., missense variants; truncated proteins, i.e., nonsense variants; or modifications in gene expression levels, i.e., upstream gene variants. The significance of these variants warrants further investigation that includes specific phenotypic data to find possible associations via genome-wide association analyses.

## Conclusion

Here, we have investigated, for the first time, the whole autosome of dromedary camels from southeast of the Arabian Peninsula to assess their genetic diversity and search for genomic signatures of selection. A degree of geographical-wise genetic distinction with a substantial level of introgression has been observed among the dromedary populations. Based on the de-correlated composite multiple signals (DCMS) approach, candidate regions and genes with signatures of positive selection were defined. Different environmental data; such as humidity, pathogens load, temperature, altitude and salinity, are needed to investigate possible correlations between these selection signatures and the dromedaries’ adaptability to their environment. Moreover, such discoveries need to be further validated by including more diverse dromedary populations from different geographical regions and habitats in the Arabian Peninsula. An improvement of the reference genome draft would serve as an advantage to similar analyses whereby the functionality of the unplaced scaffolds can be further explored. This study represents the first milestone in developing genomic markers that can be used in designing genomic-informative breeding programs to conserve the genetic diversity of this well-adapted species and improve its productivity.

## Data availability statement

The datasets presented in this study can be found in online repositories. The names of the repository/repositories and accession number(s) can be found here: https://www.ebi.ac.uk/ena, PRJEB67314.

## Ethics statement

Ethical approval was not required for the study involving animals in accordance with the local legislation and institutional requirements because no approval was required as the research only required hair samples from the animals which were collected with minimum discomfort and with non invasively.

## Author contributions

MA: Conceptualization, Funding acquisition, Writing – original draft, Writing – review & editing. AA: Data curation, Formal Analysis, Investigation, Software, Writing – original draft. ZM: Data curation, Formal Analysis, Investigation, Methodology, Software, Validation, Visualization, Writing – review & editing. FA: Conceptualization, Data curation, Investigation, Project administration, Writing – original draft, Writing – review & editing. WA-M: Funding acquisition, Writing – review & editing. SA-H: Validation, Writing – review & editing. MA-A: Funding acquisition, Writing – review & editing. HB: Conceptualization, Writing – original draft, Writing – review & editing.

## Footnotes

^1^ http://broadinstitute.github.io/picard/index.html
